# Building Blocks of Artificial CRISPR-Based Systems beyond Nucleases

**DOI:** 10.3390/ijms24010397

**Published:** 2022-12-26

**Authors:** Andrey A. Kuzmin, Alexey N. Tomilin

**Affiliations:** Institute of Cytology, Russian Academy of Sciences, Tikhoretsky Ave. 4, 194064 St-Petersburg, Russia

**Keywords:** synthetic biology, CRISPR, genome engineering, transcription, multiplexing, gRNA modifications

## Abstract

Tools developed in the fields of genome engineering, precise gene regulation, and synthetic gene networks have an increasing number of applications. When shared with the scientific community, these tools can be used to further unlock the potential of precision medicine and tissue engineering. A large number of different genetic elements, as well as modifications, have been used to create many different systems and to validate some technical concepts. New studies have tended to optimize or improve existing elements or approaches to create complex synthetic systems, especially those based on the relatively new CRISPR technology. In order to maximize the output of newly developed approaches and to move from proof-of-principle experiments to applications in regenerative medicine, it is important to navigate efficiently through the vast number of genetic elements to choose those most suitable for specific needs. In this review, we have collected information regarding the main genetic elements and their modifications, which can be useful in different synthetic systems with an emphasis of those based on CRISPR technology. We have indicated the most suitable elements and approaches to choose or combine in planning experiments, while providing their deeper understanding, and have also stated some pitfalls that should be avoided.

## 1. Introduction

For nearly 50 years, researchers have been successfully manipulating the genomes of mammalian cells. In 1974, the first transgenic mouse was obtained, opening the possibility of changing not only the genetic composition of mammalian cells but also of an entire organism [1]. In 1979, experiments were performed on transgenesis with a payload by introducing the viral *Thymidine Kinase* (*TK*) gene and selection in HAT medium [2]. Over time, it has been learned how to control cell fate in a similar way, from MyoD-induced transdifferentiation to reprogramming of somatic cells into iPSCs and beyond [3,4,5]. Now it is possible not only to perform simple transgenesis but also to create artificial chromosomes and carry out complex genome editing manipulations, taking great advantage of bacterial CRISPR systems [6,7]. Using these systems, it is now possible not only to make local changes in the nucleotide sequence of the genome but also to control gene expression, change chromosome topology, label genomic loci and individual RNA molecules, and perform other manipulations. First-order CRISPR tools have evolved into even more advanced approaches, such as DNA-based logic gates, synthetic gene circuits, multiplex genome engineering, and information recording [8,9,10,11]. In order to create such a variety of new applications, a number of genetic elements and tools have been discovered and developed. The more we learn about them, the more opportunities will emerge to integrate them into new systems. In addition, the more effectively they can be used, the more convincing such systems will be. Much current attention has been paid to effectors, such as Cas9 or Cas12a, including their standard functionality and modifications. This topic has been reviewed in many works, often overlapping among themselves [12,13,14]. However, effectors are only one aspect of complex systems that can be assembled. Other elements include, first of all, promoters and terminators, which deploy and shape the entire system. Second, elements that allow the generation of complex systems by combining many elements into a single unit, so called multiplexing tools. Third, modifications of gRNA sequences that can increase the efficiency of their function or give them additional properties. These unheeded elements are in focus of this review, which will be explored and discussed below.

## 2. Promoters

**Figure 1:**
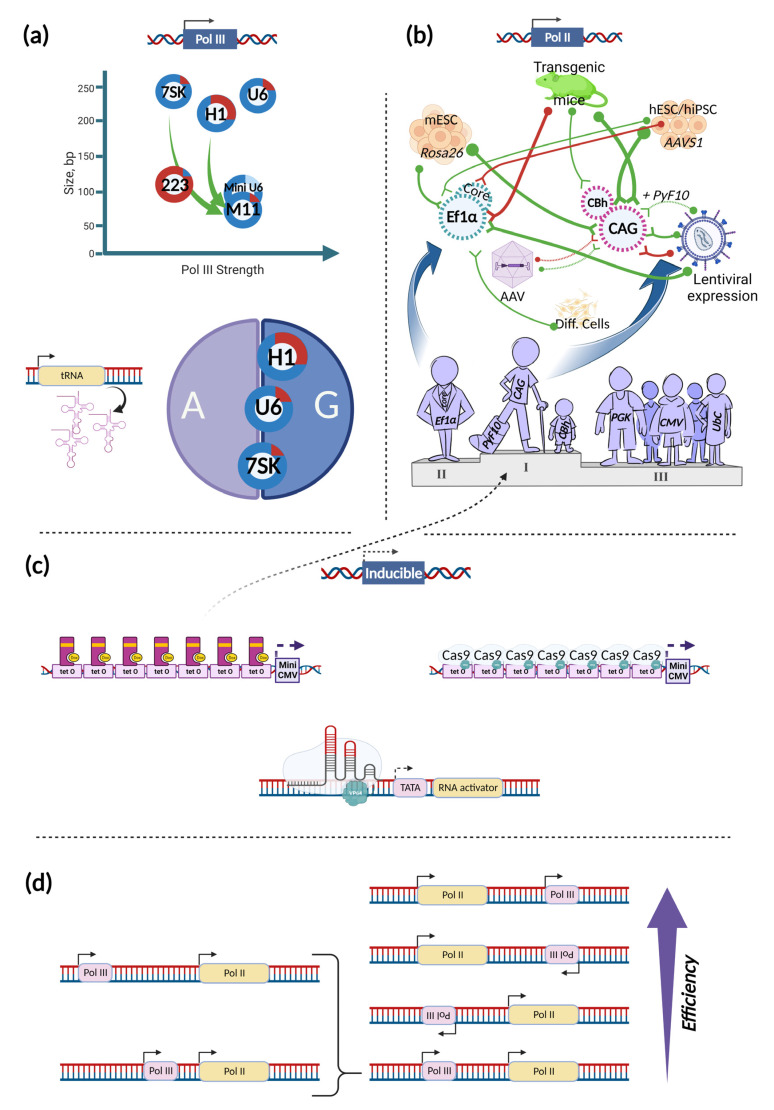
Promoter selection guide. (**a**) Pol III promoters are used mainly for Pol III-driven gRNA expression (blue sectors), but also can direct Pol II-dependent transcription (red sectors). The light blue sector for Mini U6 indicates that its Pol II activity has not been assessed. The size of the sectors reflects promoter activities. H1, U6, and 7SK position within the A/G circle indicates the importance of the corresponding nucleotide (A or G) in the position of transcriptional activation (+1). tRNA promoter, as an option for Pol III-driven transcription is depicted separately on the left of the A/G circle; (**b**) Pol II promoters vary in their activities depending on context. Green and red neuron-like connections summarize previous cases of successful (green) and unsuccessful (red) use of indicated promoters in different cell, vector, and application contexts. Line thickness reflects the amount and consistency of relevant literature for each of the cases; (**c**) Three examples of inducible promoters, including the conventional tet-ON promoter, its CRISPR-modification, and fully synthetic compact inducible promoter that is based on TATA box, RNA activator and CRISPR-activator binding site. The dashed arrow indicates the ability of the inducible promoter to drive the expression of the transgene on the level comparable to the best Pol II promoters; (**d**) Summary of the positional effect of Pol III and Pol II promoters on editing efficiency. A brace indicates variants with different distances between the promoters. Figure was created using BioRender.com.

Choosing the right configuration of promoters and terminators is one of the crucial points in the generation of an efficient, stable, and predictive CRISPR system. Promoters, usually used for driving the expression of gRNAs and transcripts encoding effectors and accessory proteins, can be divided by their activity into Pol II or Pol III promoters. Naturally, these promoters are responsible for the transcription of different types of RNA. While Pol II promoters initiate transcription of different protein-coding mRNAs, Pol III promoters are used in cells to express short RNAs, such as tRNAs, 5S rRNA, and U6 snRNA. Their basic elements (though their presence is optional), except the TATA box, also differ. BREs (TFIIB recognition elements), Inr (the initiator), MTE (motif ten element), and DPE (downstream core promoter element) elements have been characterized for Pol II promoters, whereas TFIIIB binding sites (also known as BREs), A box, and B box—for Pol III promoters [15,16].Typically, U6, H1, and 7SK promoters are used in mammals as Pol III promoters; for expression of different gRNAs, U6 and H1 are most commonly used [17]. However, their ability to drive expression of small RNAs is not exclusive. With varying efficiency, they can also drive the expression of long transcripts via Pol II (Figure 1a) [18]. The H1 promoter possesses the strongest Pol II activity, approximately six-fold stronger than U6 and even three-fold stronger than the SV40 promoter. Among these promoters, the most Pol III- specific is 7SK. To make activity of these promoters more selective, engineered versions of Pol III promoters were generated. To maximize Pol II activity, the TATA-box within these promoters was mutated, abolishing Pol III activity. This approach seems to be universally applicable because Pol II activity is significantly increased in all three cases and can be used to build compact CRISPR systems that utilize short Pol III promoters with Pol II activity to drive expression of effectors. However, information regarding their activity compared to known strong Pol II promoters in different contexts is needed. Another engineering approach is reversal, involving truncation and mutation of Pol III promoters to abolish Pol II activity and maximize Pol III-specific activity [19]. For the H1 promoter, this has been achieved by leaving only a 99-bp core sequence upstream of the transcription start site (TSS) together with the introduction of 7SK elements to this core region. As a result, the H1–7SK hybrid M11 promoter, apart from profoundly reduced Pol II activity, showed maximal Pol III activity, which was even stronger than that of the parental H1 promoter and comparable to that of the U6 promoter [19]. For the U6 promoter, a shorter version has also been generated (although without the aim to deactivate Pol II activity), showing a level of disruption of the *TCRαβ* gene similar to that of constructs based on the wild-type U6 promoter [20]. Another point to consider when using Pol III promoters is nucleotide preference at the +1 position of the TSS (Figure 1a). For the promoters described above, favored nucleotides are A and G, with the G a priori recognized to be the best choice. In fact, difference between A and G at the +1 position are insignificant in terms of the efficiency of small RNA expression. Only in terms of start site usage difference between those nucleotides appear. Specifically, for the H1 promoter, an A in the +1 position results in equal efficiency of transcriptional initiation from the –3, –2, and –1 positions, which may add undesired nucleotides to the gRNA. In the case of G, transcription initiation is shifting to the –1 position. There was no such a problem for the 7SK promoter, and for the U6 promoter an A at the +1 position shifted the TSS usage by only 12.7 percent [21]. Another reason why TSS can be altered is variability of the Pol III promoter sequence itself. It was shown for the mouse U6 promoter that sequence modification upstream of the putative initiation site leads to the TSS shifting [22].

When using Pol II promoters to drive the constitutive expression of effectors and auxiliary proteins, the main points to consider are strength, versatility, and stability. One of the most popular promoters, known for its strength and its ability to maintain high activity in the vast majority of cell types and tissues, is the CAG promoter (also erroneously referred to as CAGGS, CAGG or pCAG) (Figure 1b) [23,24,25,26]. The original CAG promoter consists of the CMV IE enhancer fused with the chicken *β-actin* promoter and the first intron, 3′-splice sequence of which has been replaced with one derived from the rabbit beta-globin gene [27,28]. Despite its popularity, the CAG promoter is not always the ideal choice. For example, in the work of Chen and colleagues, it was indeed shown that the CAG promoter was the best choice when placed in the *Rosa26* safe harbor locus in mouse ES (Embryonic Stem) cells [29]. In contrast, Tchorz et al. showed that the CAG promoter was only second in its ability to drive high level of transgene expression from *Rosa26* locus in mouse ES cells [23,29]. At the same time, mice derived from ES cells carrying a CAG-driven transgene showed strong expression of this transgene in most tissues except liver, fat, and kidney thus demonstrating CAG as a preferable promoter to drive transgene expression in long-term experiments [23]. As for human safe harbors, there have been no extensive comparisons of promoters; however, the CAG promoter in the context of the *AAVS1* safe harbor locus showed stable expression of genes of interest in hESCs, hiPSCs, and their derivatives [30,31,32,33,34,35,36]. The controversy regarding CAG promoter usability in published sources began when viral vectors were used for transgenesis. For example, in some studies CAG promoter was found to be not the ideal choice in the context of adeno-associated virus (AAV) vectors [37,38], while other studies suggested the opposite [39,40]. Another example of inconsistency has come from experiments with lentiviral vectors. Xia and coauthors showed that in human ESCs, CAG-driven EGFP was expressed poorly in the context of lentiviral vectors, being suppressed in a promoter-dependent manner [41]. Similarly, CAG promoter activity was assessed during mouse ESC differentiation into neurons. Undifferentiated ESCs and embryoid bodies transduced with CAG–GFP lentiviruses showed approximately 2.5-fold lower mean GFP intensity compared to those transduced with lentivirus carrying EF1α promoter-driven GFP. However, in fully differentiated cells, the promoters showed relatively comparable results [42]. There are further examples of strong and stable expression from the CAG promoter in lentivirus-based experiments. Ramezani et al. showed that CAG and EF1α promoters drove strong, uniform, and stable GFP expression in transduced cultures of the human hematopoietic stem/progenitor cell line KG1 [43]. In another paper, Quin and coauthors performed a comprehensive comparison of different promoters within lentiviral vectors and showed that the EF1α and CAGG promoters are consistently strong in all cell types, while in some cell types one of these two promoters tends to be slightly stronger or weaker than the other [25]. Overall, when promoter length is not crucial, the CAG promoter, even though showing above-mentioned weaknesses in viral contexts, may be considered as the best choice. The second-best choice may be the EF1α promoter mentioned above (Figure 1b). The EF1α promoter produced the highest EGFP fluorescence when stably integrated into the *Rosa26* locus in mouse ESCs, although its activity in transgenic mice is remarkably weaker than that of CAG promoter and *β-actin*-inserted control (ActB) [23]. In addition, EF1α was second best (and comparable to CAG) when driving *Fluc* expression from transiently transfected ESC and demonstrated the third-best result for *Fluc* expression from the *Rosa26* locus in the sense orientation [29]. Furthermore, EF1α was the best choice when stably integrated via linearization and electroporation in self-renewing mouse ESCs, as well as following their differentiation [44]. Finally, in several studies EF1α was reported to be advantageous in the context of lentiviral vectors used to transduce mouse and human cells, including ESCs [25,41,42,43,45,46]. The most notable drawback of the EF1α promoter is that it is not optimal for transgene expression from the *AAVS1* safe harbor locus in human iPSCs [35,36]. However, this observation is true only for the EF1α core promoter (or EFS) without intronic sequence, as a longer version of EF1α was successfully used to stably express AtAFB2-mCherry from the *AAVS1* locus for efficient, auxin-inducible degradation of proteins in human cells [47]. Furthermore, the full length EF1α promoter functions well within the *AAVS1* locus in hESCs during long-term culture and differentiation [48]. Another drawback lays into poor expression from EF1α promoter in transgenic mice [23,49].

Apart from promoters themselves, there are modifications that can be introduced to further optimize transgene composition in terms of length or to enhance expression efficiency. For example, the addition of the polyoma virus mutant enhancer *PyF10* to the CAG promoter can dramatically improve transgene expression from lentiviral vectors [26]. In addition, a shorter version of the CAG promoter, named CBh, has been developed; this version is approximately twice smaller but is still capable of driving strong and stable expression of GFP in mouse tissues [24].

As pointed out above, there is no promoter that can be considered universally ideal. The choice of such promoters should be based on vector and cell contexts; still, some controversial results even in the same context must be taken in account. With some exceptions, CAG and EF1α stand out from other promoters and can be considered, respectively, as first and second choices when planning experiments.

Constitutive expression is not always required; in some cases, expression of the effector must be induced at a precise moment in time or in a specific cell type. In these cases, inducible promoters, such as TRE [50,51], are often used (Figure 1c). It has been shown that in different cell types, the TRE promoter is comparable to or only slightly weaker than strong, constitutive promoters [25]. However, this is not the only way to induce transgene expression, especially for CRISPR-based purposes. A more compact and convenient expression driver in the context of the usage of Cas-activators consists of only a TATA box adjacent to sgRNA binding sites for transcription initiation and an RNA activator domain for efficient translation [52]. This implementation is especially relevant when Cas-activators are already included inside engineered systems. This approach can also be merged with the component of TRE promoters—minimal CMV, or more advanced, TRE3G promoter, which can accelerate the resulting potential of activation. Such experiments have been performed in which activation of TRE promoter was mediated via dCas9–VP48 (inactive Cas9 fused to 3×minimal VP16 activation domain), guided by sgTetO. The resulting activation of tdTomato expression was of almost the same efficiency as the positive control (rtTA transactivator) [53]. However, induction from the TRE promoter, mediated by Cas-activator, can be even higher. VP64 as the activation domain, representing 4 × minimal VP16, acts as the weakest activator, compared to VPR or strategies involving tethering of the activators to the sgRNA or Cas9, such as SAM or Suntag [54,55]. Deploying effectors built on those activation domains can accelerate expression from inducible promoters; expression could be even stronger than that from constitutive promoters, such as CAG. A similar approach can also be implemented to boost the expression of tissue-specific promoters with a minimal level of off-target expression [56].

Apart from these promoters, the T7 promoter is worth mentioning. It is very useful for in vitro applications, such as CRISPR-based biosensing and microinjections, and can be combined with the abovementioned promoters, for example with the U6 Pol III promoter [57,58].

The position and orientation of promoters are also important, especially for complex synthetic genetic instruments. Most papers dedicated to this problem and connected to CRISPR technologies emphasize the importance of the precise orientation and position of the Pol II and Pol III promoters when used inside a single vector (Figure 1d). Thus, it was shown that U6–gRNA(1), separated from the EFS by another U6–gRNA(2), enhanced its ability to produce indels compared to the scheme in which U6–gRNA(1) had been placed directly upstream EFS promoter. Meanwhile, the percentage of indels mediated by U6–gRNA(2) decreased when the gRNA(2) was used as a separator [59]. In another paper, Levy and colleagues found that displacing the *U6–gRNA* from the position upstream of the promoter-SaCas9 portion of the AAV cassette in the forward direction to a position downstream of this portion in the reverse direction substantially improved editing rates [60]. Furthermore, in cells transduced with AAV vectors, editing rates were significantly reduced for the U6–gRNA placed in the reverse direction downstream of the CMV promoter driving Cas9 expression when compared to constructs with the gRNA in the forward direction. In the case of the reverse orientation, transcription of both SaCas9 and guide RNA was reduced [61]. Bidirectional promoter (BiP) systems which have been developed for genome editing in plants worth particular attention. Ren and colleagues showed that with the help of BiP from rice (referred to as OsBiP1) 75.9–93.3% editing efficiency can be achieved. Such a design is interesting not only in terms of promoter strength (in fact this promoter is weaker than the unidirectional ZmUbi promoter) or simplicity, but because it can lead to an optimal effector/gRNA molar ratio [62,63]. Though it is only true on RNA level. In addition, this effect can be achieved using single promoter together with multiplexity tools which will be covered below [64].

Finally, transfer RNA (tRNA) promoters stand apart of those described above (Figure 1a). The tRNAs represent a highly conserved class of small, non-coding RNA molecules that undergo specialized steps of cleavage and posttranscriptional modification [65]. For purposes of CRISPR-based systems, tRNA gene promoters have been used to replace U6 promoters for gRNA production via Pol III. For example, the expression of gRNA from 70-bp promoters from MHV68 tRNA and glutamine tRNA showed comparable levels of luciferase activity inhibition compared to the U6 promoter [66]. This approach can be used as an alternative to small versions of U6 or H1 promoters for compact expression systems.

## 3. Transcriptional Termination and Polyadenylation Signals

**Figure 2:**
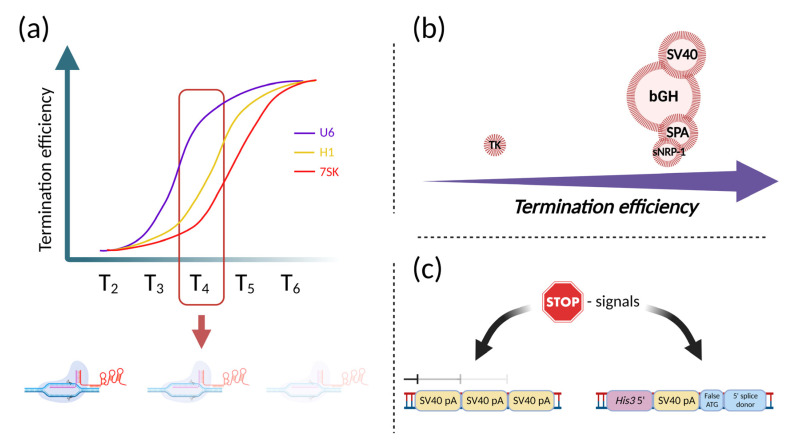
Picking a right transcriptional terminator. (**a**) Schematic representation of termination efficiencies for different Pol III promoters. Each curve represents the dependency of termination on the length of the poly-(T) sequence (T_n_). The red frame indicates critical for multiplexing T_4_-stretch, naturally presented in Cas9 sgRNA. Faded Cas9/sgRNA/DNA complex represents consequences of the T_4_ -stretch inside Cas9 sgRNA on multiplex transcription from Pol III promoter; (**b**) Schematic comparison of the termination efficiencies from different termination signals. The diameter of the circle represents the relative size of the terminators. The termination efficiency was equally high for most of the termination signals, clearly except for TK; (**c**) STOP-signal variants. Left—STOP signal made with the aim to maximize termination efficiency, combining three SV40 poly(A) signals. Right—STOP signal which not only terminates transcription but also prevents translation. This STOP signal consists of a C-terminal sequence of the yeast His3 gene, SV40 polyadenylation signal, and a false translation initiation signal, combined with a 5′ splice donor site. Figure was created using BioRender.com.

Terminators and Pol III termination signals represent another essential basic issue to consider for the rational engineering of efficient CRISPR-based systems. Termination of Pol III transcription requires a specific signal—the poly-(T) sequence (T_n_) on the non-template strain of transcribed DNA [67]. Recently, Gao and colleagues determined the termination efficiency of T-stretches of different lengths in conjuncture with the 7SK, H1, and U6 promoters. They showed that termination efficiency for U6 promoter was equal to 75% for T_4_ and 95–100% for termination signals longer than T_5_. For the 7SK and H1 promoters, same termination efficiency was observed for T_5_ and beyond. However, for these promoters, termination was also observed using T_4_, with efficiency of 27% and 18%, respectively [68]. This is very valuable information for designing multiplex systems, in which many gRNAs are expressed from a single promoter. This especially applies to spCas9 applications, even in non-multiplex formats, because the spCas9 gRNA contains a T_4_-stretch within the gRNA scaffold sequence (Figure 2a). A solution to this problem has been found by flipping the A–T base pairs inside this T_4_-stretch [69]. Another major concern regarding Pol III termination relates to the usage of Cpf1 (Cas12a). As it was previously shown, an additional U-tail inhibits the activity of the AsCpf1-crRNA complex. This issue should also be considered in the AsCpf1-based systems [68].

For Pol II-driven transcription, many terminators have been used. Most popular are SV40, bGH and TK polyadenylation signals [70]. They differ by two main parameters—efficiency of termination and length. In terms of efficiency, the SV40 late polyadenylation signal is similar to bGHpA, but it is almost half as long, which is important for the development of compact synthetic systems [71]. The rbGlob poly(A) signal is another highly efficient terminator derived from the rabbit *beta-globin* gene. Based on this poly(A) signal, a short synthetic terminator has been developed—SPA (synthetic poly[A] site), which is only 49 bp in length [72]. Another compact poly(A), even shorter than SPA, was obtained from the soluble *neuropilin-1* (*sNRP-1*) gene poly(A) signal. Of only 32 bp in length, it showed a similar level of generated mRNA compared to the SV40 polyadenylation signal (Figure 2b) [73]. Of note, apart from efficiency in mRNA production, poly(A) signals can also affect vector stability when transiently transfected into cells [74].

To maximize the efficiency of termination for Pol II-mediated transcription, repeated poly(A) signals are used, consisting of trimerized SV40 polyadenylation signals and is often referred to as the STOP signal (Figure 2c) [75]. It has been shown that a single or bipartite STOP signal does not show the same efficiency of blocking downstream expression as that of the original, tripartite signal [76]. However, if undesired elongated RNA is unlikely to interfere with potential synthetic systems or their parts functioning on the RNA level, it can be concluded that a strong STOP signal is unnecessary in this case. Another reason to not use tripartite STOP signals in RNA-based systems is that this is useless in terms of efficiency of synthesis of the RNA of interest, because repetition of poly(A) sequences did not produce an increase in the amount of RNA [77]. However, this can be useful to prevent unspecific protein synthesis, which can have an impact even in the presence of small levels of undesired RNA, especially if strong promoters are used. In summary, to make a system specific in terms of protein synthesis, the addition of the STOP signal may be the ideal choice; however, if the system’s functioning depends on the RNA, it may be unnecessary. A different approach can also be used, in which the STOP signal not only blocks transcription but also simultaneously terminates transcription and prevents translation [78,79].

## 4. Multiplexity

The main goal of this paper is to assemble information that will help researchers find the proper way to design and assemble complex, CRISPR-based systems and related components. Indispensable parts of such systems have been discussed above. This section now describes the multiplexing and fundamental principles that have been established in this field, as well as the genetic elements that are able to join different parts of complex systems inside a single unit and functionally divide them at certain stages.

**Figure 3:**
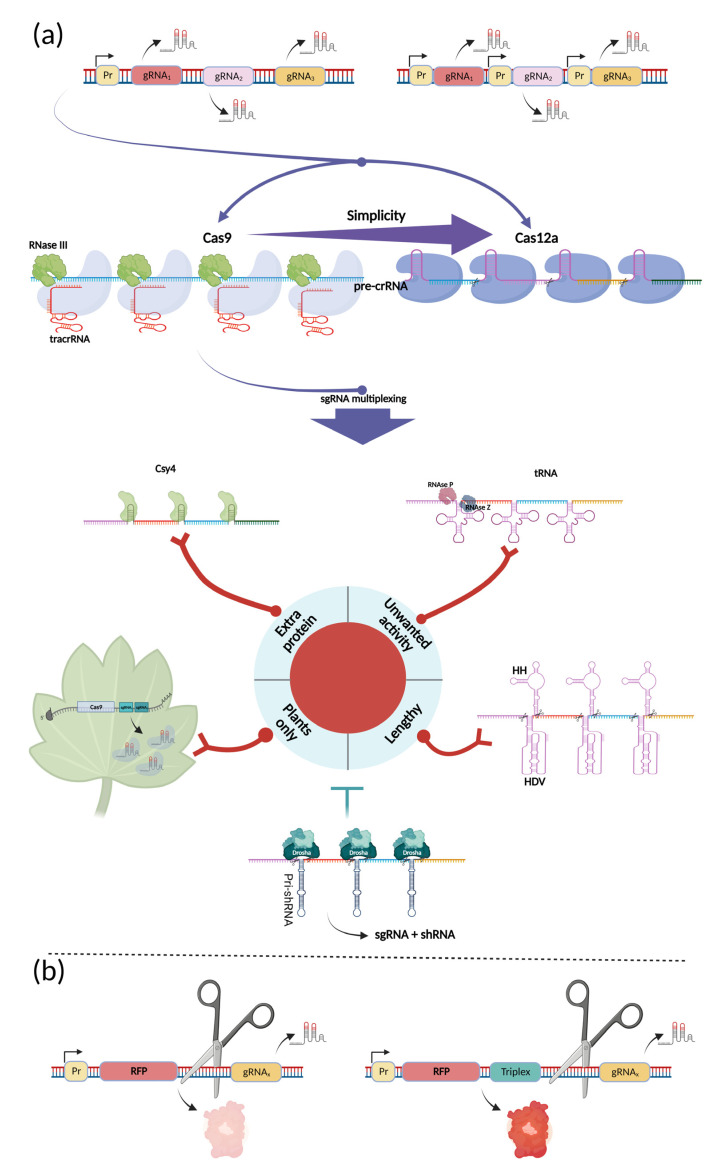
Multiplexing expression modules. (**a**) Different approaches of multiplexing have been developed for CRISPR-based applications. In general (top), the expression of multiple gRNAs can be provided by a single promoter or multiple promoters. In the first case, multiplexed gRNA arrays can be processed naturally by different mechanisms: for Cas9, pre-crRNA is processed by the Cas9/RNase III/tracrRNA complex, while for the Cas12a-based systems, processing of pre-crRNA is simpler and is mediated by the single effector. As for Cas9, a deployment of alternative multiplexing strategies is important when sgRNAs are used. These strategies include Csy4-mediated cleavage, tRNA-mediated RNaze P/Z processing, ribozyme-mediated cleavage, native sgRNA processing in plants, and shRNA-based strategy. Red neuron-like connections depict the limitation of each Cas9-multiplexing approach, when applicable; (**b**) A triple helix RNA (named triplex) protects RNA from degradation, stabilizing its 3′end, thus allowing further effective translation. Figure was created using BioRender.com.

Multiplexity for CRISPR systems often refers to a multiplicity of gRNAs expressed within a single assembly. Expression in this case can be mediated by a single promoter or from several promoters at one time (Figure 3a) [80]. To allow the expression of many gRNAs from a single promoter with a desired function, mechanisms of RNA processing must be utilized. Fortunately, Cas effectors can process corresponding gRNA arrays in different ways. In type II-A, B, and C-based systems, spCas9 mediates RNase-III-dependent crRNA processing by its binding in conjunction with tracrRNA [81,82,83]. This feature of CRISPR machinery was used in the initial work that presented its functionality inside mammalian cells even without bacterial RNase III co-expression [84]. Currently, type-II CRISPR systems in which crRNA is combined with tracrRNA in the single guide RNA (sgRNA) are used. For the multiplication of many spacers, alternative tracrRNA-independent solutions have been used [85]. Another popular CRISPR-effector, type V Cas12a (or Cpf1), is also capable of processing its own crRNA array. Apart from Cas9, Cas12a crRNA does not require tracrRNA for assembling of functional gRNA, which naturally exists as a single polyribonucleotide. In addition, there is no need for RNase III; to process every crRNA, as Cas12a possesses an RNase domain. This is very convenient in terms of multiplexity because additional elements are not required. It is also functional, as additional processing units can lower the effectiveness of the entire system. Because RNA processing is mediated via a separate domain in Cas12a, it is possible to use dCas12a for multiple gene regulation [86,87].

Despite the potential of type-V systems to create multipurpose multiplex systems, Cas9-based applications are currently most common. In such approaches, multiplexity, due to the predominant absence of tracrRNA, is achieved in four different ways: through the use of ribozymes, tRNA (with P and Z RNase processing), Csy4, or through expression from individual promoters. Comparisons of the performance of these systems have been made earlier [85]; this review focuses on a deeper description of the advantages and disadvantages of these approaches, as well as on cases of their most appropriate deployment (Figure 3a).

Among these approaches, the most processive one is based on the CRISPR-associated protein Csy4 (Cas6, Cse3) which belongs to type I CRISPR systems [88,89]. It has been shown that using this approach, it is possible to simultaneously express eight functional gRNAs from a single Pol II promoter. Moreover, editing frequencies of these gRNAs increased when expressed from the strongest promoter and reached 25–30% for each individual gRNA in the system, with median activity that even surpassed that of individual gRNAs expressed from the U6 promoter. In addition, it was shown that the 20-nucleotide (nt) recognition site of the Csy4 protein rather than the canonical motif of 28 nucleotides works most efficiently [90]. Csy4 itself can be considered as a limitation of this approach; its use may be undesirable if the number of proteins in the system is limited—for example if there is a decrease in the efficiency of expression of one of the proteins in the polycistronic cassette [91]. Apart from this issue, its ability to produce multiple gRNAs from a Pol II promoter with fairly uniform editing frequencies seems to be unlimited; nevertheless, further investigation is needed.

The next approach is based on the pre-tRNA that is naturally processed by RNases P and Z [92]. It has been shown in yeast that their use can allow the expression of six different gRNAs from a single Pol III promoter with almost perfectly equal disruption efficiency. However, with an increase in the number of gRNAs to seven, disruption efficiency began to vary greatly and declined strongly when the number of gRNAs was eight [93]. Of note, similar behavior was observed with the expression of multiple gRNAs from the Pol-III promoter using Csy4 [90]. In the case of Csy4, it was the seventh and eighth guide RNAs at which working efficiency dropped. This weakness can be explained by repetitive poly-T tracts that act as termination signals for Pol III, as mentioned above. The use of the Pol-II promoter in combination with tRNA would possibly be ideal for uniform and highly multiplexed gRNA expression. In this case, the largest disadvantage lies in the pre-tRNA length of 77 bp, which is almost four times longer than the Csy4 motif. Additionally, as previously stated, tRNAs have promoter activity, which is not desirable if different gRNAs must be activated from different time points. However, promoter activity of tRNAs can be avoided by their re-engineering [94].

The third popular approach is the use of HDV and HH ribozymes [95]. Considering that both flanking ribozymes are needed for proper cleavage of premature gRNA, their size is even larger than the size of a single tRNA: 43 nucleotides for the HH ribozyme (excluding its 6-nt part shared with gRNA) and 68 nts for the HDV ribozyme. Thus, there are 111 nts in sum, which is 1.5 times more than tRNA. Another disadvantage of these ribozymes is the approximately twofold-lower efficiency of gRNA expression compared to tRNA [96].

In addition to these basic approaches, other approaches exist that have not received wide application. The first of these relies on native sgRNA processing, without the involvement of additional ectopic factors, and has been performed only in plants. The key factors in this process are spCas9 and intracellular RNases. This approach has been used to produce one or more functional sgRNAs from shared Cas9 mRNA [97,98,99].

Another is not well known but is a promising approach based on the use of the cellular RNA interference system. This approach has been tested in mammalian cells. In this case, the gRNA sequences are separated by the pri-shRNA (approximately 70 bp) and form a single transcript synthesized by the Pol II polymerase. During the processing of the primary mRNA, it is cleaved in the nucleus by a multiprocessor complex, which includes proteins such as Drosha and Pasha, and is further processed by intracellular RNases to form a functional gRNA. In turn, shRNAs are transported into the nucleus and may additionally participate in the interference with the mRNA of interest or act as a scramble control [100,101]. In addition, siRNA can be theoretically used to regulate the gRNA sequences themselves.

It is also worth mentioning that multiplexing has been achieved not only by the co-expression of different gRNAs. It is possible to effectively express mRNA, part of which consists of processible gRNA and part is further effectively translated into functional protein. This is possible due to the addition of an RNA structure called a triplex, which protects cleaved RNA from degradation (Figure 3b) [86,102,103].

Thus, a wide range of possibilities exist to express many gRNAs in the cell simultaneously. Each of these approaches has its advantages and disadvantages and can be chosen based on the goals and limitations of the experiment.

## 5. gRNA Modifications

This review seeks to provide guidilines in designing experiments that are based on the intracellular expression of CRISPR systems components. Therefore, chemical types of gRNA modification, which have been reviewed elsewhere [96,97,98], are not discussed in deep. Nevertheless, we shall point out two cases in which chemical modifications are especially useful. The first case is the protection from nucleases, when different chemical modifications of the gRNA termini are purposely introduced [104,105]. Another case of chemical modifications regards conjugates between gRNA and donor DNA or between Cas9 and oligonucleotides that recruit donor DNA. It has been shown by Lee and coauthors, that gRNA and donor DNA conjugates can enhance HDR frequency approximately threefold compared to free gRNA and donor DNA when transfected via polycations [106]. In turn, Ling et al. demonstrated that recruitment of the unmodified donor DNA to the Cas9-Oligonucleotide conjugate substantially increased HDR efficiency in both human cell culture and mouse zygotes [107].

Returning to the natural, gRNA-coding, sequence alterations, one should stress that in general, all manipulations of gRNA sequences can be classified into two main types: enhancing modifications and modifications that expand the functionality of gRNAs. Be-low, we will describe these main types of gRNA modifications and provide their deeper understanding in terms of their usage and from a fundamental point of view.

### 5.1. Sequence Modifications

**Figure 4:**
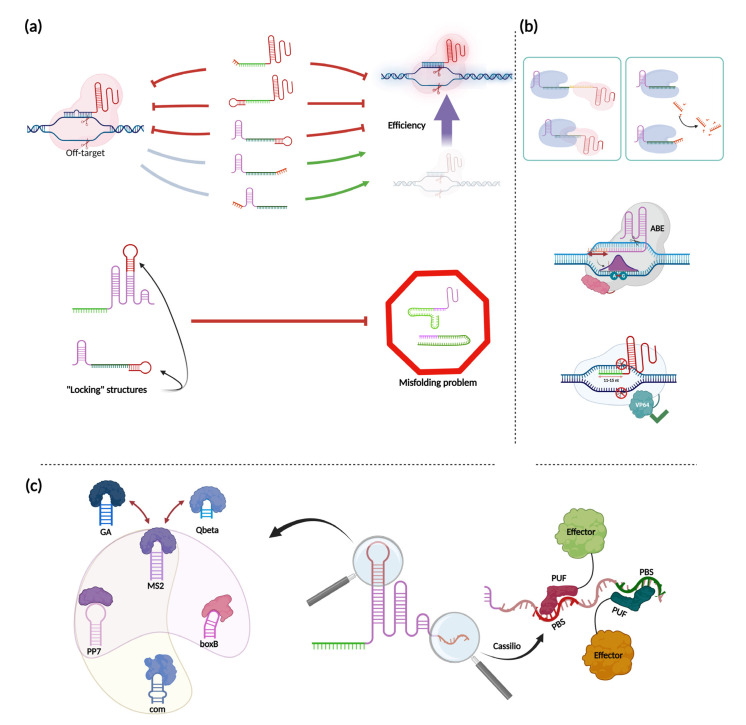
gRNA modifications. (**a**) Impact of sgRNA and crRNA modifications on OFF-target activity and editing efficiency. Adding hairpin structures or small flaps to the end of gRNAs results in different outcomes for Cas12a- and Cas9-based systems. Red inhibitory lines depict the antagonism between different modifications and the OFF-target effect or editing efficiency. Green arrows depict the opposite. Gray lines mark modifications that do not have any effect on specificity. The bottom scheme depicts the approach to block misfolding of impaired gRNA by introducing a “locking” structure inside their sequences; (**b**) Different functional consequences of gRNA sequence modifications. Upper left: a shared usage of single gRNA by Cas12a and Cas9. Upper right: an enhancement of trans-cleavage activity of Cas12a used in pair with elongated crRNA. Middle: restriction of the modification window for the Cas9 base-editor ABE for spacers truncated to 19 nts or elongated to a length of more than 24 nts. Bottom: a decrease in the length of sgRNA to 15 nts or fewer (but not less than 11 nts) completely eliminated the ability of Cas9 to cleave DNA while retaining the ability to bind recognition sites and to activate thereby adjacent genes; (**c**) Implementing sequence augmentations and aptamers. On the left side, different RNA aptamers are depicted. Dark red arrows indicate interchangeability between aptamer binding proteins. Transparent colored clouds depict examples of non-overlapping multiple usage of different aptamers inside shared synthetic systems. The right side depicts a part of the Cassilio system, with effectors fused with PUF-domains, specifically programmed to bind different PBS (PUF-binding site). Figure was created using BioRender.com.

In general, all modifications of the nucleotide sequences of sgRNA or crRNA can be located either at the ends of gRNA or inside hairpin structures (Figure 4a). Regarding the end modifications, attempts have been made to either extend or reduce the gRNA length to potentially enhance the specificity of Cas9 or Cas12 effectors. For Cas9, it was shown that the addition of two unpaired guanines at the 5′ end of gRNA (GGX_20_) reduced off-target effects, but this modification could also greatly reduce the efficiency of specific cleavage of different targets [108]. Similar impairments have been shown in other work that accurately assessed the mechanics and domain specificity of such modifications. Mullally et al. showed that two or three unpaired guanines at the 5′ end of the spCas9 gRNA significantly reduced the level of DNA cleavage. For stCas9, this decrease in the efficiency of DNA cleavage was RuvC-domain-specific and did not affect the NHN-nuclease domain (this information can be considered for the experiments involving the use of nickases). In addition, the dynamics of formation of the R-loop almost did not change. However, for the “GGG” case, the dissociation time was increased, thus stabilizing R-loop formation [109]. Furthermore, the addition of a 20-nt hairpin to the 5′-end of gRNA led to the formation of a partial hyperstable R-loop, inhibiting further DNA cleavage [109]. These data suggest that such 5′ modifications are not so desirable if increasing the efficiency of double-stranded DNA cleavage is important and may require an extensive selection of suitable guides; however, they can be very useful for regulation of gene expression when nuclease-inactive Cas9 is used.

An alternative approach, also involving a hairpin designed at the 5′ end, has been proposed by Kocak et al. Instead of using an additional hairpin hanging from the 5′ end, the authors made it complementary to the spacer sequence [110]. They showed that this modification increased the specificity of Cas9 cleavage but did not increase the level of activation (and even slightly decreased it in some cases) with the dCas9-p300 system, which would not have been expected based on the data described above. Moreover, the presence of Cas9 on off-target sites was not reduced compared to wild-type gRNA. Thus, there are some contradictions between those works. However, it should be kept in mind that Kocak et al. used a model system based on HEK293T cells transfected with Cas9 and guide plasmids. Given the predisposition of these cells to transfection and the high level of expression of the transfected constructs, it can be assumed that the activation efficiency reached a plateau; the result may have been different if basal activation level was lower. This paper also demonstrated a similar effect on the specificity of nuclease activity for an artificial hairpin structure at the end of the AsCas12a and LbCas12a crRNA spacers. Due to the structural features of Cas12a crRNA, in this case the hairpin was at the 3′ end. Similarly, this modification reduced the activity of Cas12a crRNA at the ON-target sites, though this reduction was clear only for the SaCas12a. In another study, Bin Moon et al. showed that the addition of three or four Us or a 4T(A)6T motif (*A* is placed to avoid generating a STOP signal) to the 3′-end of the crRNA significantly increased the efficiency of AsCpf1 and LbCpf1. For AsCpf1, the mean increase for the U-rich configuration was 2.31-fold. At the same time, this did not affect the off-target effects [111]. Interestingly, these findings disagree with another work, which has been mentioned earlier [68]. Additionally, elongation of the 5′ end of crRNA LbCas12a, FnoCas12a, and AsCas12a increased the editing efficiency in HEK cells, without altering specificity of Cas12a. For example, for AsCpf1, 5′ extension of crRNA +4 to +25 nucleotides enhanced gene editing efficiency by approximately twofold in HEK cells [18,112]. This result is surprising because the 5′-end of crRNA is naturally processed due to the RNase activity of Cas12a itself. On the other hand, additional cleavage by Cas12a can be seen as the hypothetical reason of such enhancements hidden in natural processing of crRNA. Apart of those steric reasons, such additions may inhibit gRNA degradation by intracellular RNases before they bind to Cas12a.

Another explanation of such effects of Cas12a crRNA spacer end elongation, resulting rather in improvement of crRNA functioning than in its inhibition, can reside in the ability of some of these modifications to prevent gRNA misfolding. Creutzburg and co-authors demonstrated this effect for additional 3′-end hairpin structures. The authors specifically stress the importance of such modifications in their general guidelines for the design of Cas12a-associated crRNAs, which focus on the tracking of misfolding at the 5′end of crRNA and their resolution. In addition, they emphasize the importance of monitoring the absence of undesirable secondary structures for multiplex RNA constructs [113]. Not surprisingly, Cas9-based systems face the same gRNA misfolding problem, and this problem was solved in a similar way. Riesenberg et al. have described several variants of gRNA misfolding that can lead to reduced modification rates for Cas9. Among them is the annealing of the spacer to itself or to the 5′-end of the gRNA and even inversion with respect to the tracrRNA if the tracrRNA is used separately. The authors solved the problem of misfolded gRNA by introducing a super-stable hairpin inside the first hairpin of gRNA, locking the folding structure of the entire gRNA. In eight out of 10 cases, this modification increased the efficiency of genomic editing, contributing to the correct folding of gRNA [114]. Apart from these articles, it is worth mentioning the work done by Kweon et al., where the authors not only extended the 5′- and 3′-ends of the spCas9 sgRNA and the Lb/AsCas12a crRNA, respectively, but also combined these gRNAs to form a common spacer. Experiments in HeLa and HEK293T cells have shown the effective use of such constructs, resulting in either no change in indel frequency compared to individual gRNAs or a decrease of approximately 10–20 percent. At the same time, such modifications did not affect the off-target effects. Moreover, the authors showed the possibility of creating gRNA with double spacer sequences, which can be used for multiplex applications [115].

The elongation of gRNA sequences may have other functional consequences (Figure 4b). For example, it was shown that LbCas12a increases its trans-cleavage activity due to the elongation of the 3′-end, while the elongation of the 5′-end does not result in this effect [116]. Another unusual effect of elongation of the spacer of the gRNA (more than 24 nts) is the restriction of the modification window by the Cas9 base-editor ABE to the eighteenth nucleotide from the 3′ end of the spacer. The same effect was also shown when the spacer length was reduced to 19 nucleotides [117]. Further truncation of the Cas9 sgRNA sequence to 17 nucleotides has been shown to decrease knockout efficiency by 10–20% in iPSC and MSC without decreasing off-target efficiency [118]. Though this data does not agree with previous experiments, conducted on human U2OS cells [119]. This allows us to conclude that effect of the modifications of gRNA to reduce off-target effect are context-dependent. At the same time, a decrease in the length of sgRNA to 15 or fewer nts completely eliminated the ability of Cas9 to cleave DNA while retaining the ability to bind to recognition sites and activate adjacent genes. In this case, the minimal functional sgRNA length was 11 nts [120,121]. Other modifications of Cas9 sgRNA have been implemented on the upper stem and the first hairpin of sgRNA in the form of additional aptamer sequences, which are discussed below.

Coming back to Cas12a, it is worth mentioning another type of modification, namely division of the crRNA into two separate pieces, resulting in the formation of a split version. In this case, information regarding the effect of such modifications is rather controversial. For example, in Jurkat cells, ErCas12a (MAD7) was shown to be tolerant to splitting at position 2, 3, 4, and 5 with a maximum impairment in indel efficiency of approximately 25% [122]. On the other hand, applying the split crRNA for AsCas12a in HEK cells completely abrogated cleavage efficiency [123]. Thus, clear evidence that split crRNA can be used with different Cas12a orthologs will require further extensive investigation. Additionally, if design of the system suggests that two parts of gRNA should be expressed separately, the usage of Cas9 or its orthologs is a best choice. Though the type V-B Cas12b/C2c1 can be seen as alternative to Cas9 in this case [124].

### 5.2. Sequence Augmentation, Aptamers

In order to add extra functions or properties to CRISPR systems, so-called RNA aptamers, which can tether specific proteins and bind them, have been used (Figure 4c) [125,126]. The most common aptamer used is the viral MS2 hairpin, which can be bound by MS2 coat protein (MCP) and placed into gRNA as an addition to natural hairpin structures inside. The main applications of MS2 are related to the activation of genes due to the fusion of various chromatin modifiers to MCPs, as well as to the fluorescent labeling of certain regions of the genome or mRNA [127,128]. Other hairpins exist besides MS2, for example PP7 and boxB. Such additional hairpin sequences not only provide an alternative but also allow different aptamers to be combined into one system. For these purposes, it is important that the aptamer-ligand pair be highly specific and not overlap. This is true for MS2, PP7, and boxB, as they were used together for simultaneous imaging of up to six chromosomal loci in individual live cells [129]. An example of a poor choice for multiplexing two or more aptamers is the combination of GA and Qbeta aptamers, as GA protein and Qbeta binding protein not only bind to their own hairpins but also have affinity for the MS2 hairpin [130]. In addition to the aptamers described above, there is also a com aptamer, which binds to the Com protein and has been used simultaneously with MS2 and PP7 to create complex transcriptional programs [131].

An additional parameter to consider when selecting aptamer hairpins (if their performance has not been characterized in cell cultures of interest) is their origin. If they are naturally present in the cells of interest, the entire working system can be affected and undesirable side effects can be obtained. For example, U1a hpII (hairpin structure in U1 snRNA), which is normally found in human cells and binds to the small nuclear ribonucleoprotein (snRNP)-specific protein U1A (also known as SNRPA) would be better used in non-human cells, for example in yeast [132].

The Cassilio system is worth mentioning separately. This approach can dramatically increase the number of available RNA-binding proteins that can be associated with gRNA [133]. This approach is based on Pumilio proteins. These are RNA-binding proteins that contain the Pumilio/FBF (PUF) RNA-binding domain which can be programmed to bind a specific octamer RNA sequence (the PUF-binding site, PBS) [134]. Modifications within the PUF domain make it possible to program its specificity for various RNA octamers, which in turn can further expand the possibilities for creating complex systems based on RNA-binding proteins [135].

## 6. Conclusions

The use of Cas9 (Cpf1, etc.)-based methods can be simple and follow the “one cell—one event” principle, for example by knocking out a given gene or inserting a transgene by homologous recombination. The second category is associated with multiple or sequential, dependent actions. For example, it is necessary to drive complex cell differentiation via synthetic gene circuits based on Cas-activators. This implementation can be multiplex, time-dependent, and event-driven. All elements must work effectively, often in a continuous, precise, and predictable manner. For this purpose, it is essential to understand not only how effectors work but also how to assemble all the elements of the system. Relevant information appears at times, but unfortunately it often remains implicit, lagging work performed in other areas. Starting with promoters, there is a large number of variants to pick from. It is not difficult to choose them by their size or to choose from Pol-III promoters; however, information regarding Pol-II promoters is more complicated. A promising approach is to create and test a variety of synthetic promoters that can be small and highly effective. Similarly, not many studies have been performed to compare the efficiency of different polyadenylation signals in different cell types, but most of them seem to be equally effective; thus, it is better to choose these elements by size if compactness is crucial and to combine them and other elements when precise transcriptional and translational regulation are needed.

Regarding multiplexity, there are also many tools to use. Here, information is more profound, and it is not difficult to choose the right element suitable for user needs. However, it is worth stressing the importance of developing and using Cas12a-based multiplex systems because of Cas12a build-in multiplexing properties. As for different modifications, there is a large amount of information regarding sequence modifications that can increase the stability of gRNA or modify its properties. Although information regarding aptamers or gRNA stabilization is straightforward, specific modification of gRNA with the aim to reduce off-target effects may be too laborious. As for other modifications inside sgRNA scaffolds or tracrRNA sequences, which are not related to the stabilization of gRNA structure, the most promising for Cas9 is the A-U flip, which prevents premature termination [69]. This modification, together with the stabilization of crRNA structure, may be considered universal in enhancing genome editing or other CRISPR-based applications. Other modifications inside scaffold sequences are rather empirical and do not have fundamental explanation [136]. As for Cas12a crRNA scaffolds, it was shown that stem sequence mutants had reduced genome-editing activities, so this region was recognized as conserved [137]. However, modifications inside the loop sequence of the 5′-handle are allowed and can be implemented to enhance genome editing activity [137,138]. Coming back to specificity, it seems that a better way to reduce off target effects, if these are an issue, is to use modified high-fidelity effectors or their alternatives [139,140]. Another way to overcome possible off target effects is the usage of nucleases with different PAM requirements. For example, for Cas9 nuclease, several mutants, which broaden a number of editable genome sites have been developed [141,142,143]. These alternative sites allow picking less off target prone sites, unattainable by conventional Cas9. An ultimate solution for the off target issue can be found in selecting from repertoire of nucleases including the recently developed minimal-PAM Cas9 enzyme SpRY Cas9. This effector recognizes NRN PAM and allows to perform genome editing almost at any desired point of the genome [143,144]. Though its basal fidelity is not ideal, further modifications may address this issue. Overall, correct navigation through the vast number of different synthetic units will result in many possibilities for the creation of different, new, complex, CRISPR-based systems, which in turn will lead to a new methods and applications.

## Data Availability

Not applicable.
